# A numerical investigation of the effect of surface wettability on the boiling curve

**DOI:** 10.1371/journal.pone.0187175

**Published:** 2017-11-10

**Authors:** Hua-Yi Hsu, Ming-Chieh Lin, Bridget Popovic, Chii-Ruey Lin, Neelesh A. Patankar

**Affiliations:** 1 Department of Mechanical Engineering, National Taipei University of Technology, Taipei, Taiwan; 2 Department of Electrical and Biomedical Engineering, Hanyang University, Seoul, Korea; 3 Department of Mechanical Engineering, Northwestern University, Evanston, United States of America; Institute of Materials Science, GERMANY

## Abstract

Surface wettability is recognized as playing an important role in pool boiling and the corresponding heat transfer curve. In this work, a systematic study of pool boiling heat transfer on smooth surfaces of varying wettability (contact angle range of 5° − 180°) has been conducted and reported. Based on numerical simulations, boiling curves are calculated and boiling dynamics in each regime are studied using a volume-of-fluid method with contact angle model. The calculated trends in critical heat flux and Leidenfrost point as functions of surface wettability are obtained and compared with prior experimental and theoretical predictions, giving good agreement. For the first time, the effect of contact angle on the complete boiling curve is shown. It is demonstrated that the simulation methodology can be used for studying pool boiling and related dynamics and providing more physical insights.

## 1 Introduction

Boiling occurs in a variety of industrial applications such as high heat flux electronic devices [1], chemical processes [2], power plants [3], etc. During boiling, a heated surface is adjacent to a liquid, which vaporizes. The large latent heat of vaporization makes it an efficient mode of heat transfer in the nucleate boiling mode. Boiling is quantified in terms of a plot of heat flux versus the wall superheat defined as the temperature of the wall minus the saturation temperature (i.e. the boiling point) at the pressure of the liquid. The heat flux divided by the wall superheat is the heat transfer coefficient (HTC). Large HTC is an indication of efficient heat transfer.

There are various modes of boiling. During nucleate boiling, vapor bubbles form at a superheated surface which rise up in the liquid. Eventually, the heat flux reaches a maximum, which is called the critical heat flux (CHF). Increasing the superheat of the heated surface beyond the CHF value leads to drastically reduced HTC. This is because more vapor with lower thermal conductivity accumulates near the surface, eventually forming a stable film in the film boiling mode. In pool boiling applications, such as electronic equipment cooling, the drastic reduction in boiling heat flux after CHF may lead to devastating results.

Significant enhancement of HTC during boiling has been reported for porous surfaces [4], and surfaces with carbon nanowires [5, 6], silicon [7], and copper [7, 8]. In all cases, it was found that there is significantly more vapor bubble nucleation in the presence of nanoscale roughness compared to smooth surfaces. Another factor that affects boiling heat transfer is wettability, which is the ability of a liquid to wet (or be spread over) a solid surface [9, 10]. It is known that wettability can change the temperature at which CHF occurs or the temperature at which the transition of film boiling to nucleate boiling occurs, i.e. Leidenfrost point. Surface characteristics are critical to determine the efficiency of heat transfer. Surface properties such as roughness and wettability can alter the transition temperature from the nucleate boiling to the film-boiling regime. Experiment has revealed that hydrophobic surface brings out high critical heat flux (CHF), and hydrophobic surface benefits bubble nucleation. Increasing the energy efficiency by tailoring optimal surfaces could profoundly impact many industrial applications. However, contact angle effect always comes with surface roughness effect and it is difficult to distinguish from one to the other.

Boiling process involves complicated physics of phase change. With the present technology and measuring equipment, it is still difficult to observe the real dynamics of interface phase change. However, experiment has captured the temperature distribution along the liquid-vapor phase boundary [11, 12]. As for numerical parts, there are only limited numbers of numerical studies on phase change since it is a multi-scale physics involved problem which still remains a great challenge using existing tools and the drastic density change between liquid and vapor within less than microscale interface causes numerical difficulties.

Several simulation methods have been proposed to study the vapor-liquid phase change problem, such as the molecular dynamics (MD) simulation [13], lattice Boltzman method (LBM) [14–16], immersed boundary method [17], direct front tracking method [18, 19], level set (LS) method [20], and the volume-of-fluid (VOF) method [21, 22]. Numerical simulation of boiling [23–25] and specifically calculation of the boiling curve from computations [15, 16, 26] have been reported.

The objective of this study is to perform numerical simulations of boiling phenomena to investigate the effect of contact angle (CA) or wettability on boiling heat transfer using a volume of fluid (VOF) method combined with a static contact angle model. The primary focus is on reproducing qualitative trends in the whole boiling curve using one single model and gaining more physical insights into the underlying mechanisms. In this work, numerical simulations were performed for three-dimensional cylindrical and two-dimensional planar unsteady laminar flow of incompressible liquid and vapor. This was also the first time the effect of contact angle is shown in the complete boiling heat transfer curve.

## 2 Numerical method and modeling

In this work, the VOF method is chosen since the fluid mass can be conserved appropriately and it can be applied on a larger scale with any grid compared with the LBM and MD method. The continuing work can be extended to a multi-bubble problem to predict real industrial phase change applications.

### 2.1 Governing equations

In the VOF method, the two phases are represented by phase volume fractions such that

$$\begin{array}{c} \alpha_{l}+\alpha_{v}=1, \end{array}$$ (1)

where *α* is the volume fraction, subscripts *l* and *v* represent the liquid and vapor phases, respectively. The governing equations consist of the continuity equations for the two phases and a one-fluid model for the momentum and energy conservation equations.

$$\begin{array}{c} \left\{ \begin{array}{c} \frac{\partial }{\partial t}\left(\alpha_{v}\rho_{v}\right)+\nabla \cdot \left(\rho_{v}\alpha_{v}\vec{u}\right)={\dot{m}}_{lv},\\ \frac{\partial }{\partial t}\left(\alpha_{l}\rho_{l}\right)+\nabla \cdot \left(\rho_{l}\alpha_{l}\vec{u}\right)=-{\dot{m}}_{lv},\\ \frac{\partial }{\partial t}\left(\rho \vec{u}\right)+\nabla \cdot \left(\rho \vec{u}\vec{u}\right)=-\nabla p+\nabla \cdot \left(\mu \nabla \vec{u}\right)+\rho \vec{g}+\vec{F},\\ \frac{\partial }{\partial t}\left(\rho C_{p}T\right)+\nabla \cdot \left(\rho \vec{u}C_{p}T\right)=-\nabla \cdot \left(k\nabla T\right)+S_{h}, \end{array}\right. \end{array}$$ (2)

where *ρ*_*v*_ and *ρ*_*l*_ are the vapor and liquid densities, respectively, *t* is the time, $\vec{u}$ is the average fluid velocity, and ${\dot{m}}_{lv}$ is the mass source due to liquid to vapor phase change. In the momentum conservation (third) equation, *p* is the pressure, $\vec{g}$ is the gravitational force, and *ρ* and *μ* are the density and viscosity of the mixture of liquid and vapor, respectively. In the energy conservation (fourth) equation, *T* is the temperature, *C*_*p*_ is the mixture specific heat, *k* is the mixture thermal conductivity, and *S*_*h*_ is the heat source. *ρ*, *μ*, *C*_*p*_, and *k* are:

$$\begin{array}{c} \left\{ \begin{array}{c} \rho =\rho_{l}\alpha_{l}+\rho_{v}\alpha_{v},\\ \mu =\mu_{l}\alpha_{l}+\mu_{v}\alpha_{v},\\ C_{p}=C_{pl}\alpha_{l}+C_{pv}\alpha_{v},\\ k=k_{l}\alpha_{l}+k_{v}\alpha_{v}, \end{array}\right. \end{array}$$ (3)

where subscripts *l* and *v* represent the liquid and vapor phases, respectively. Along the liquid–vapor interface, surface tension results from the greater attraction force between liquid molecules than to the molecules in the vapor. Brackbill porposed a continuum surface force (CSF) model to include the surface tension effect [27], which is commonly used in the continuum VOF model. The origin of this source term can be considered from the specific case where the surface tension is constant along the interface, and where only the forces normal to the interface are considered. The pressure drops across the interface can be estimated in terms of the surface tension coefficient, *σ*, and the surface curvature as measured by two radii in orthogonal directions, *R*_1_ and *R*_2_:

$$\begin{array}{c} p_{2}-p_{1}=\sigma \left(\frac{1}{R_{1}}+\frac{1}{R_{2}}\right) \end{array}$$ (4)

Hence, in the momentum equation, $\vec{F}$ is the surface tension force between the two phases that is expressed as a volume force density:

$$\begin{array}{c} \vec{F}=\sigma \frac{\alpha_{l}\rho_{l}\kappa_{l}\nabla \alpha_{l}+\alpha_{v}\rho_{v}\kappa_{v}\nabla \alpha_{v}}{\frac{1}{2}\left(\rho_{l}+\rho_{v}\right)}, \end{array}$$ (5)

where *σ* is the surface tension and the interface curvature is given by:

$$\begin{array}{c} \kappa_{l}=-\kappa_{v}=-\nabla \cdot \left(\frac{\nabla \alpha_{l}}{|\nabla \alpha_{l}|}\right). \end{array}$$ (6)

The effect of contact angle at fluid interface in contact with solid boundary then can be estimated within the CSF model in terms of *θ*_w_ which is the equillibrium contact angle between the solid and fluid. It is a static contact angle which is measured when the fluid is at rest. If *θ*_w_ is the contact angle at the wall, then the surface unit normal $\hat{n}$ at the calculation cell is

$$\begin{array}{c} \hat{n}={\hat{n}}_{\text{w}}\cos\theta_{\text{w}}+{\hat{n}}_{\text{t}}\sin\theta_{\text{w}} \end{array}$$ (7)

where ${\hat{n}}_{\text{w}}$ is the unit vector normal to the wall and ${\hat{n}}_{\text{t}}$ is the unit vector tangential to the wall.

### 2.2 Phase-change model

**Fig 1:**
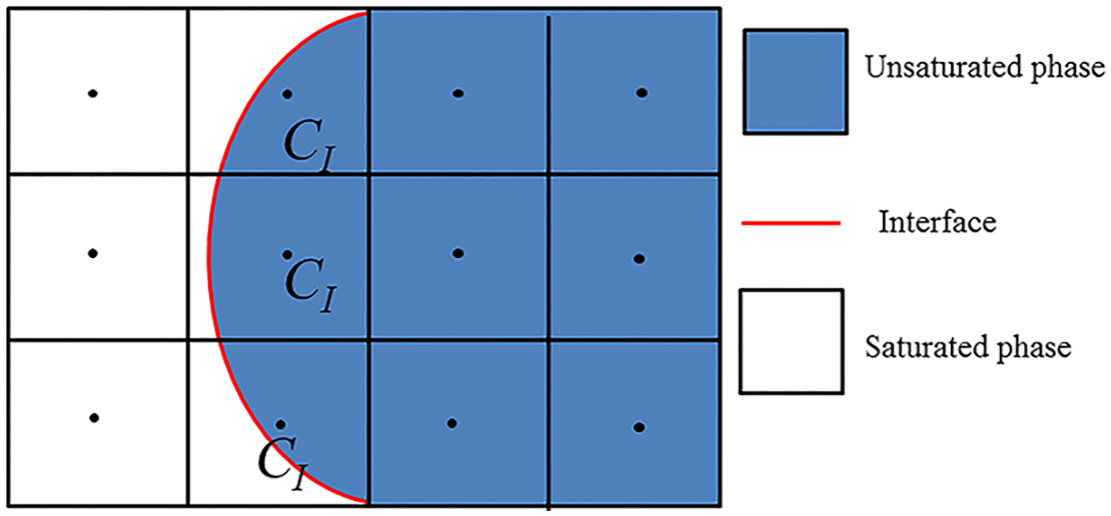
Schematic diagram of the liquid-vapor interface and the mesh in the numerical model.

The mass and energy exchange between the two phases can be governed by some phase change model coupled with the governing equations [28]. In the model used in our simulations, the thermal conductivity of the interfacial grid cells is equal to that of the unsaturated phase [29]. The interfacial heat flux *Q*_*pc*_ causing the liquid to vapor phase change is then calculated as [29]

$$\begin{array}{c} Q_{pc}=\sum_{f\in C_{I}}\text{max}\left\{\left(-k_{\text{uns}}A_{f}\nabla T_{f}\right)\cdot {\vec{n}}_{f,\text{in}},0\right\}, \end{array}$$ (8)

where *C*_*I*_’s are interfacial grid cells (see Fig 1), *f* denote faces of *C*_*I*_, *A*_*f*_ is the face area, ∇*T*_*f*_ is the temperature gradient at the cell face, and ${\vec{n}}_{f,in}$ is the unit normal vector at the cell face which points into *C*_*I*_. The mass source due to phase change at *C*_*I*_ can be calculated by:

$$\begin{array}{c} {\dot{m}}_{lv}=-{\dot{m}}_{vl}=\frac{Q_{pc}}{h_{lv}V_{CI}}, \end{array}$$ (9)

where *V*_*CI*_ is the volume corresponding to grid cell *C*_*I*_, and *h*_*lv*_ is the latent heat of vaporization. The corresponding heat source term in the energy equation is calculated as:

$$\begin{array}{c} S_{h}=\frac{Q_{pc}}{V_{CI}}\;\text{at}\;C_{I}. \end{array}$$ (10)

## 3 Problem definition

**Fig 2:**
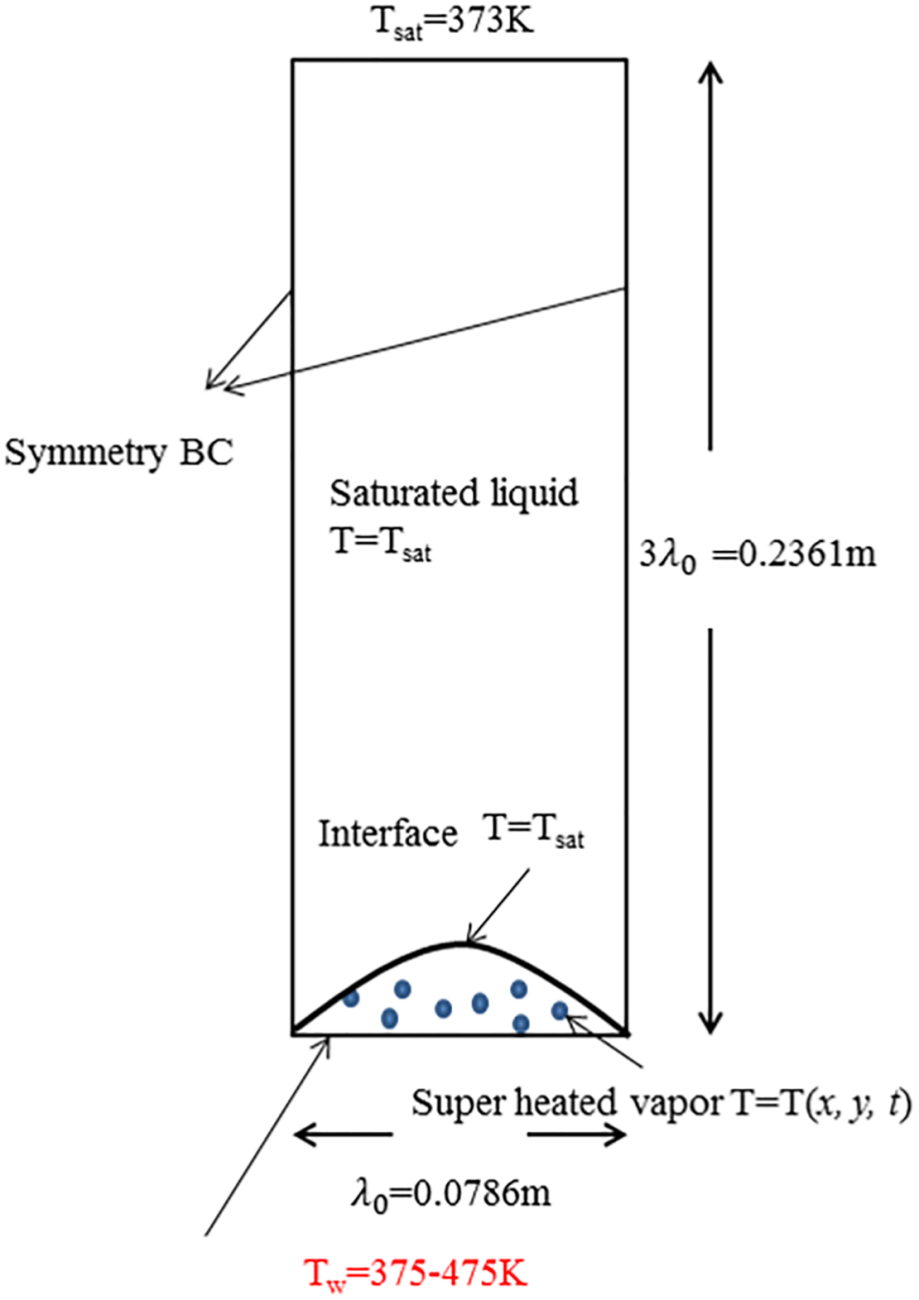
Schematic diagram of problem geometry and boundary conditions.

The primary goal is to qualitatively investigate how the contact angle influences the boiling curve, i.e., the heat transfer during various phases of boiling. Fig 2 shows the configuration used in this study. Computations were performed for three and two-dimensional, unsteady, incompressible flow with heat transfer. For the VOF method, an interface tracking method, small amount of vapor phase has to be specified at the initial of the computation [16, 25]. This is the reason why the VOF method cannot be used to study the nucleation process. Vapor phase was initially placed at the bottom wall. If the film boiling mode is not stable, then the vapor wraps into a bubble and the dynamics proceeds in the nucleate or transition boiling regime. The governing equations were solved using the finite volume method. The governing equations are solved using the software ANSYS^®^ Fluent Academic Research, Release 16.2.

### 3.1 Geometry and boundary conditions

Two dimensional simulation domain is with a width of λ_0_ and a height of 3λ_0_, and three dimensional simulation uses cylinder computational domain. λ_0_ is the Taylor-Rayleigh instability wavelength, which is calculated using the working fluid properties listed in Table 1:

$$\begin{array}{c} \lambda_{0}=2\pi {\left(\frac{3\sigma }{(\rho_{l}-\rho_{v})g}\right)}^{\frac{1}{2}}=\text{0.0786}\;\text{m}. \end{array}$$ (11)

**Table 1:** Properties of the fluid used in simulation.

Parameter	liquid	vapor
*ρ* Density (kg/m^3^)	200	5
*μ* viscosity (Pa⋅s)	0.1	0.005
*k* Thermal conductiviy (W/mk)	40	1
*C*_*p*_ specific heat (J/kg⋅k)	400	200
*h*_*lv*_ latent heat (J/kg)	1 × 10^4^	
*σ* surface tension (N/m)	0.1	

The boundary conditions are as follows. The upper boundary condition is set as a pressure outlet with temperature *T* = *T*_sat_. Symmetric boundary conditions are used for both sidewalls of the simulation domain. The bottom boundary is a no-slip superheated wall with a constant contact angle along the surface. The bottom wall has a constant temperature ranging from 2K to 100K above saturation temperature *T*_sat_ = 373K. To consider the effect of contact angle, the bottom wall has a contact angle ranging from $5^{{}^{\circ}}-180^{{}^{\circ}}$. For simplicity, static contact angles were considered as the key trends were successfully resolved by static contact angle simulations although studying the effect of dynamic contact angles was feasible [25]. Initially, a linear temperature profile from *T*_w_ at the bottom wall to *T*_sat_ at the liquid-vapor interface is specified in the vapor domain. The liquid domain has an initial temperature equal to *T*_sat_. A “microlayer” is deemed relevant for hydrophilic cases [15, 16]. In these cases, the microlayer contribution to heat transfer has been reported to be around 20% [23]. Hence following a prior work [25], and for simplicity, the microlayer was not modeled in this work [30].

The initial shape of the vapor-liquid interface is perturbed to initiate the bubble growth. The initial interface position, which is the height of vapor film from the bottom wall, is given by:

$$\begin{array}{c} y=\frac{\lambda_{0}}{128}\left(1+\cos\left(2\pi \frac{\left(x+\frac{\lambda_{0}}{2}\right)}{\lambda_{0}}\right)\right). \end{array}$$ (12)

Gravity is pointed in the vertically downward direction.

### 3.2 Fluid properties

For the purposes of obtaining better numerical stability, the density ratio of liquid and vapor cannot be large [31], so the working fluid is chosen to be an artificial one with properties listed in Table 1. For comparison, the properties of water (not the fluid in our simulations) are listed in Table 2.

**Table 2:** Properties of water.

Parameter	liquid	vapor
*ρ* Density (kg/m^3^)	1000	0.5
*μ* viscosity (Pa⋅s)	1 × 10^−3^	1 × 10^−4^
*k* Thermal conductiviy (W/mk)	0.68	0.0225
*C*_*p*_ specific heat (J/kg⋅k)	4.22 × 10^3^	2.03 × 10^3^
*h*_*lv*_ latent heat (J/kg)	2 × 10^6^	
*σ* surface tension (N/m)	0.0589	

Further comparison between the properties of working fluid and water can be done based on non-dimensional parameters. The primary non-dimensional parameters are listed below:

$$\begin{array}{c} \left\{ \begin{array}{c} Ja=\frac{\text{[specific}\;\text{heat][temperature]}}{\text{[latent}\;\text{heat]}}=\frac{C_{pl}\Delta T}{h_{lv}},\\ Pe=\frac{\text{[specific}\;\text{heat][length][velocity][density]}}{\text{[conductivity]}}=\\ \frac{C_{pl}\lambda_{0}\sqrt{g_{y}\lambda_{0}}\rho_{l}}{k_{l}},\\ Fr=\sqrt{\frac{\text{[velocity]}}{\text{[gravity][time]}}}=\sqrt{\frac{\sqrt{g_{y}\lambda_{0}}}{g_{y}\sqrt{\lambda_{0}/g_{y}}}}=1,\\ Re=\frac{\text{[density][velocity][length]}}{\text{[viscosity]}}=\frac{\rho_{l}\sqrt{g_{y}\lambda_{0}}\lambda_{0}}{\mu_{l}},\\ We=\frac{{\text{[density][length][velocity]}}^{2}}{\text{[surface}\;\text{tension]}}=\frac{g_{y}\lambda_{0}^{2}\rho_{l}}{\sigma },\\ Pr=\frac{Pe}{Re}=\frac{C_{pl}\mu_{l}}{k_{l}}, \end{array}\right. \end{array}$$ (13)

where [⋅] denotes scale of the corresponding physical variable. In the above list, *Ja* is the Jakob number, *Pe* is the Peclet number, *Fr* is the Froude number, *Re* is the Reynolds number, *We* is the Weber number, and *Pr* is the Prandtl number. Liquid properties are used in defining these parameters. Additional non-dimensional parameters are the ratios of density, viscosity, thermal conductivity, and specific heat of the two phases (liquid and vapor). These ratios can be deduced from Tables 1 and 2. The non-dimensional parameters for the working fluid in our simulations are listed below:

$$\begin{array}{c} \left\{ \begin{array}{c} J_{a}=8\times 10^{-2}\;\text{to}\;4,\\ Pe=\text{3.849},\\ Re=\text{3.85},\\ We=\text{1.023},\\ Pr=1. \end{array}\right. \end{array}$$ (14)

For comparison, typical values of the same non-dimensional parameters for water during boiling are listed below:

$$\begin{array}{c} \left\{ \begin{array}{c} J_{a}=4.22\times 10^{-3}\;\text{to}\;2.11,\\ Pe=2.36\times 10^{3},\\ Re=3.80\times 10^{2},\\ We=\text{1},\\ Pr=\text{6.21}. \end{array}\right. \end{array}$$ (15)

The Jakob number (*Ja*) represents the ratio of sensible heat to latent heat absorbed during the phase change process. The Jakob number of working fluid in simulations is larger than that of water which represents a better ability to phase change. The typical critical heat flux (CHF) for water in experiments is around 10^6^ W/m^2^; a lower CHF for the working fluid may be due to different *Ja*. The Reynolds numbers (*Re*) in both fluids are in the laminar regime. The Weber numbers (*We*), which depend on surface tension, are close to unity in both fluids. The Prandtl numbers (*Pr*) in both fluids are on the order of unity, implying that thermal diffusivity is close to momentum diffusivity.

## 4 Results and discussion

The numerical results are presented in three-dimensional cylindrical and two-dimensional planar simulations, respectively.

### 4.1 Three-dimensional cylindrical simulation

**Fig 3:**
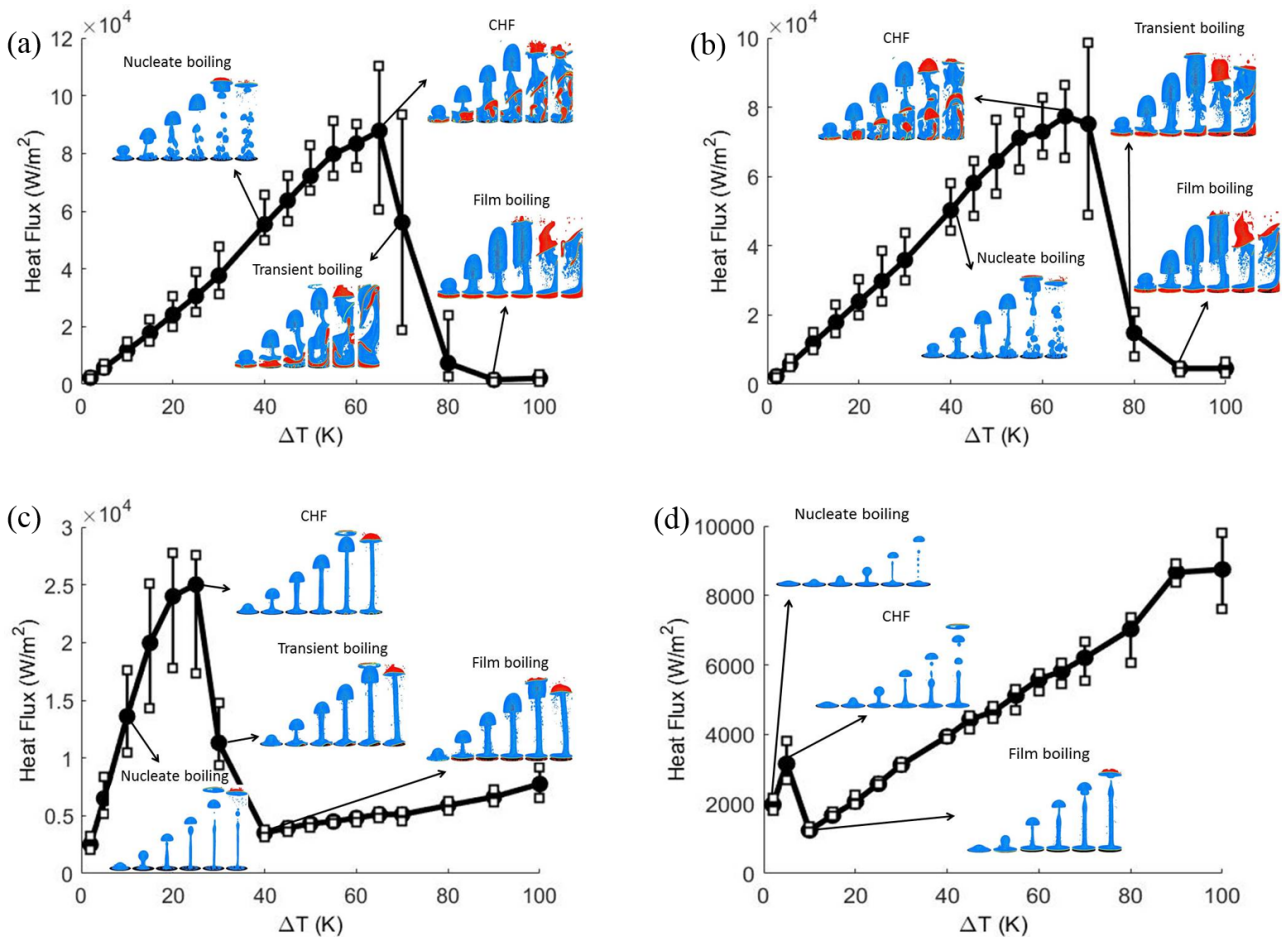
Three dimensional cylindrical simulation of boiling curve for contact angle = (a)10°, (b)60°, (c)120°, and(d)160°. Vertical bars indicate the range of temporal fluctuations in heat flux.

**Fig 4:**
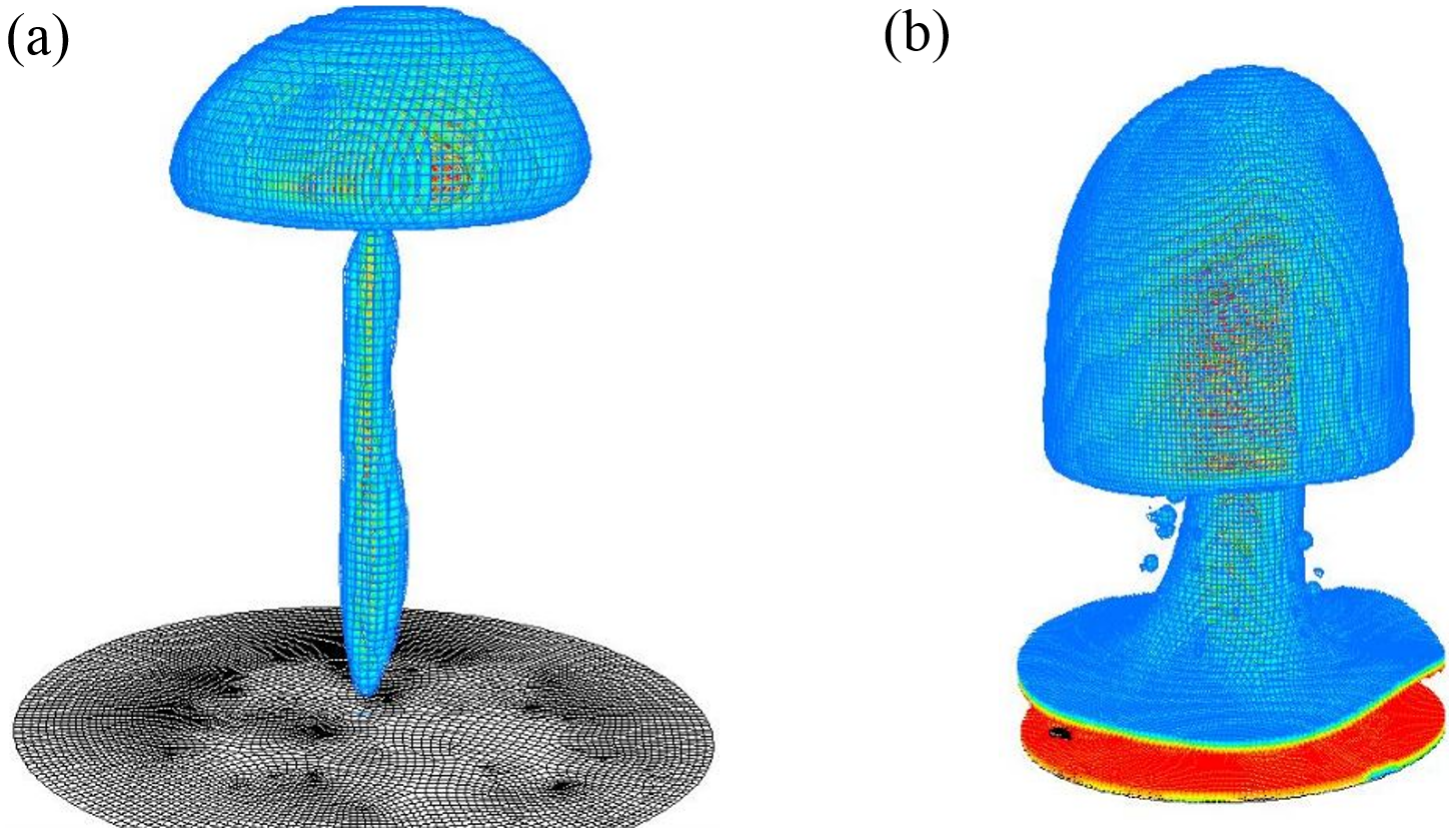
Three dimensional cylindrical simulation results of vapor phase for contact angle = 60° at (a) ΔT = 15K and (b) ΔT = 90K.

Boiling curves for the contact angle = 10°, 60° (hydrophilic) and 120°, 160° (hydrophobic) are shown in Fig 3. These figures show the effect of contact angles on the boiling curve. Vapor is initially placed next to the bottom boundary which is superheated with a contact angle. All key aspects of the boiling curve, namely nucleate boiling, critical heat flux condition, transition boiling, and film boiling are qualitatively captured in the calculated numerical results. The phase contour plots of transient vapor bubble dynamics are also shown for different superheats. Around the critical heat flux, the heat flux fluctuates significantly from instant to instant. Hence, the trends fluctuate much more. To emphasize this issue, the ranges of heat flux noises are depicted in Fig 3 with error bars. The axisymmetric cylindrical simulation is time consuming and computationally expensive. Fig 4 shows the three dimensional cylindrical simulation results of vapor phase for contact angle = 60° at (a) ΔT = 15K and (b) ΔT = 90K which show the mushroom shape. On a workstation with 4 cores of Intel^®^ XEON^®^ CPU E3-1225 v5 @ 3.3 GHz processor and 32 GB RAM, the computation time for three-dimensional cylindrical simulation is more than 12 days (288 h). However, it takes about 4 hours for a two dimensional planar simulation. Hence, the two dimensional planar simulations are carried out to determine the qualitative trend of boiling curve.

### 4.2 Two-dimensional planar simulation

The two-dimensional planar numerical results are presented in four subsections: transient bubble dynamics, boiling curve, critical heat flux (CHF), and Leidenfrost point (LFP).

#### 4.2.1 Transient bubble dynamics

**Fig 5:**
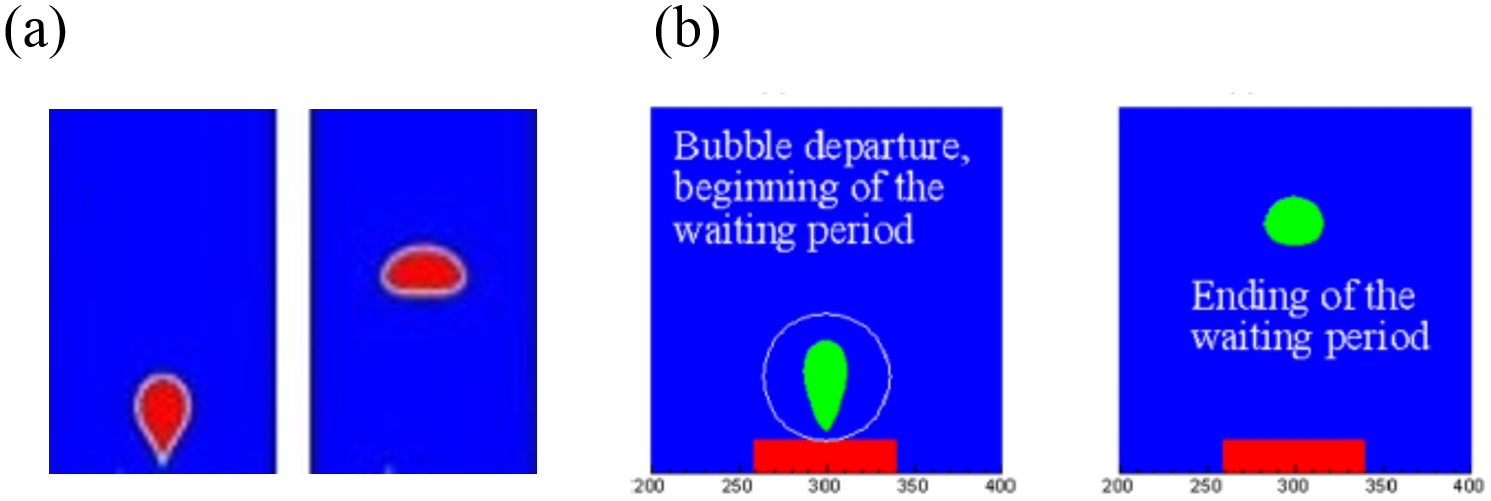
Bubble growth and departure for hydrophilic surface using (a) volume of fluid method and (b) lattice Boltzman method [16].

**Fig 6:**
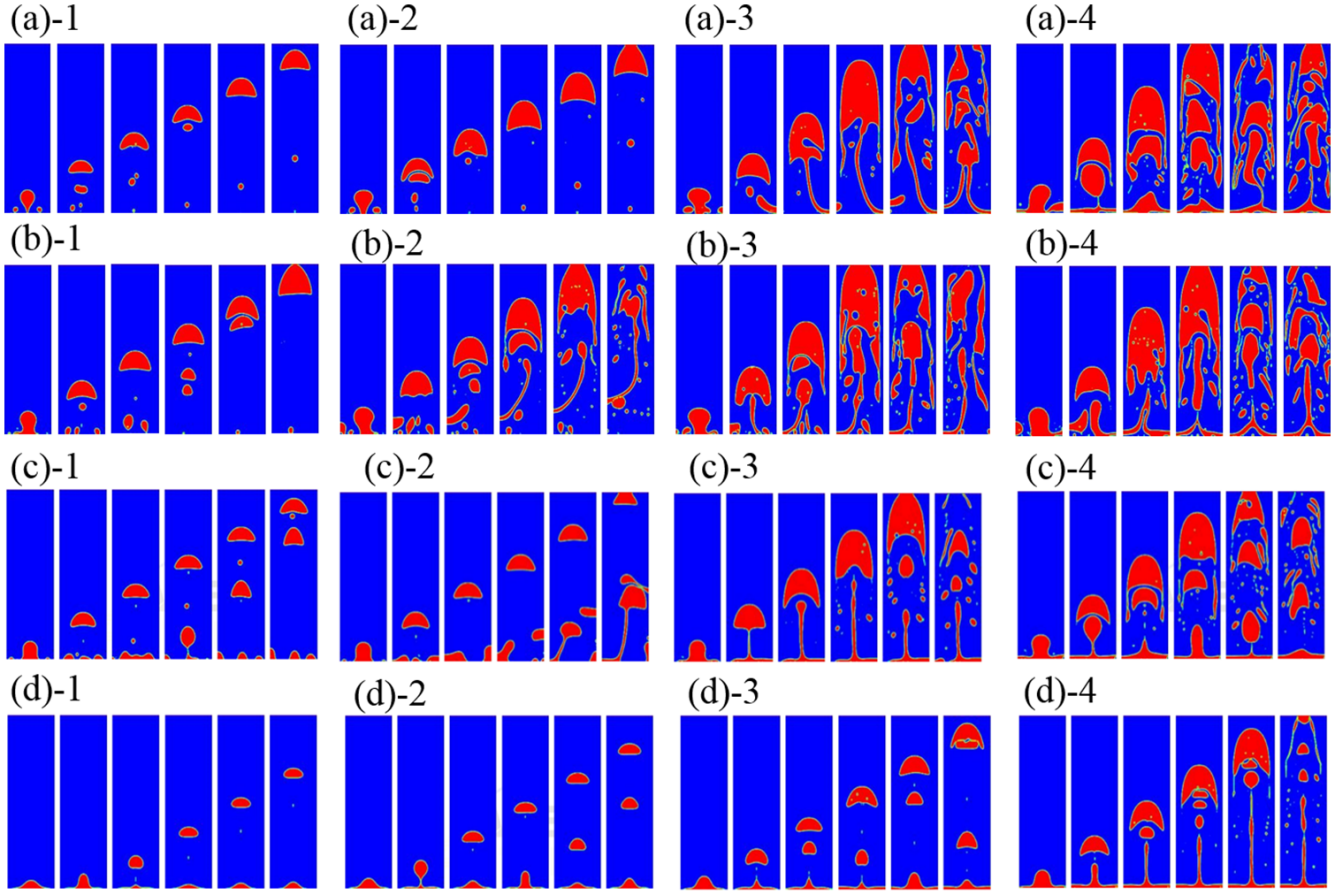
Two dimensional planar vapor dynamics at (a) contact angle = 10° (1)Nucleate boiling: vapor bubble growth at ΔT = 25K (2)Critical heat flux: vapor bubble dynamics at ΔT = 45K (3)Transient boiling: vapor bubble dynamics at ΔT = 55K (4)LFP: vapor bubble dynamics at ΔT = 60K; (b)contact angle = 60° (1)Nucleate boiling: vapor bubble growth at ΔT = 30K (2)Critical heat flux: vapor bubble dynamics at ΔT = 50K (3)Transient boiling: vapor bubble dynamics at ΔT = 55K (4)LFP: vapor bubble dynamics at ΔT = 60K; (c)contact angle = 120° (1)Nucleate boiling: vapor bubble growth at ΔT = 20K (2)Critical heat flux: vapor bubble dynamics at ΔT = 25K (3)Transient boiling: vapor bubble dynamics at ΔT = 30K (4)LFP: vapor bubble dynamics at ΔT = 40K; (d)contact angle = 160° (1)Nucleate boiling: vapor bubble growth at ΔT = 2K (2)Critical heat flux: vapor bubble dynamics at ΔT = 5K (3)LFP: vapor bubble dynamics at ΔT = 10K (4)LFP: vapor bubble dynamics at ΔT = 15K.

Vapor is initially placed next to the bottom boundary which is superheated with a static contact angle imposed. If the film boiling mode is not stable, then the vapor wraps into a bubble and the dynamics proceeds in the nucleate or transition boiling regime. The continuing bubble generated depends upon the amount of remaining vapor accumulated at the heated wall. In the transition and film boiling regimes, a new vapor bubble is formed naturally from the interfacial instability. In the nucleate boiling regime, while the surface can become fully wetted without any vapor, one can numerically introduce repeated nucleation bubbles artificially. The rate of introduction of new nucleate would depend in nucleation models that take into account the nature of the surface. This gives no difference from prior models in literature. To validate this work, this simulation is compared with the result of Lattice Boltzmann method [16]. Fig 5 shows the bubble growth and departure for the hydrophilic case. Both VOF and LBM show the entire bubble departed from the heating surface and no vapor accumulated at the solid boundary. Fig 6 shows the phase contour plots of transient vapor bubble dynamics under different superheats, i.e., nucleate boiling, critical heat flux, transient boiling, and LFP, and contact angles of 10°, 60°, 120°, and 160°, respectively. Note that contact angles less than 90° are regarded as hydrophilic, whereas those above 90° are regarded as hydrophobic. The red fluid area represents the vapor phase state while the blue area represents the liquid phase state.

(i) Hydrophilic surface:

For the contact angles of 5° − 90°, it is observed that liquid tends to wet the bottom wall more. For example, at contact angle = 10° (Fig 6(a)-1), the initial vapor layer forms a bubble and the liquid wets the wall. The vapor layer is increasingly unstable for more hydrophilic walls. In the nucleate boiling phase, bubbles are formed at the bottom wall. With an increase in the wall superheat, the vapor-production rate increases and the growth period decreases. It is seen that the bubble diameter at departure increases with wall superheat. For a fixed contact angle, the departure bubble diameter depends on the growth rate, which increases with wall superheat.

**Fig 7:**
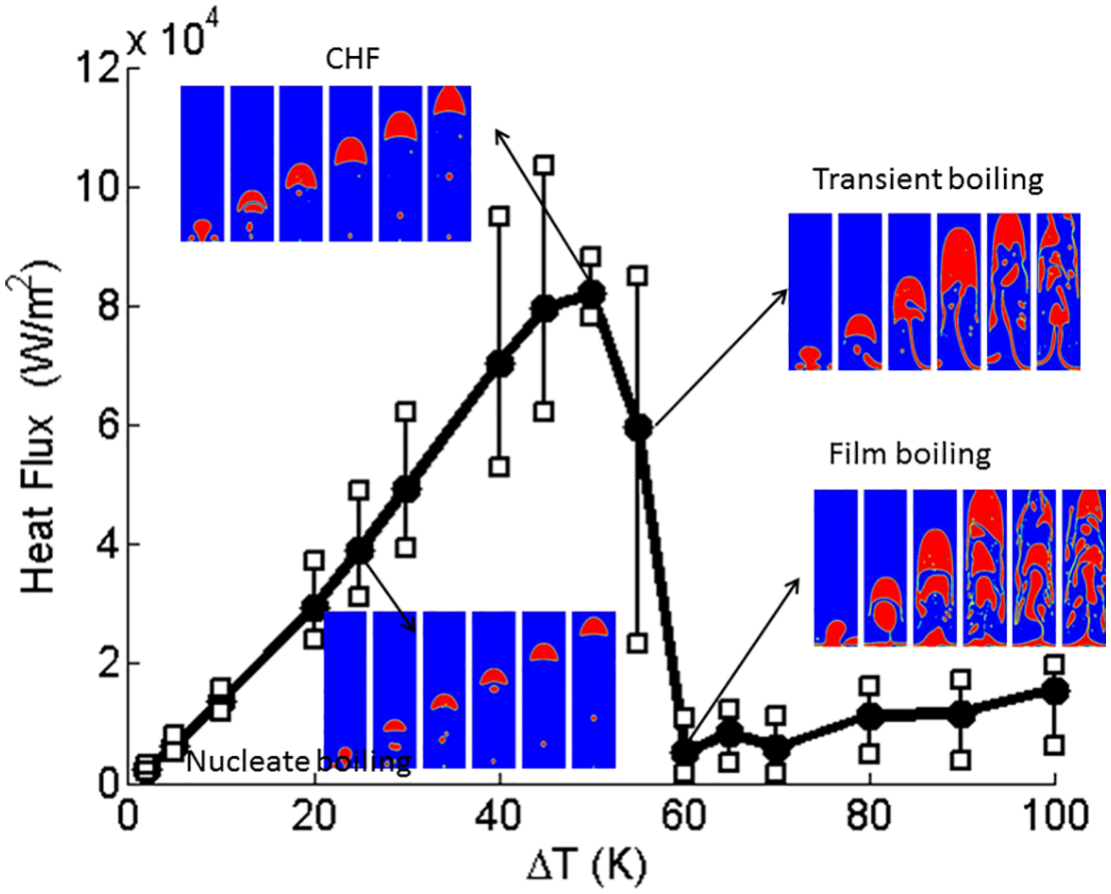
Two dimensional planar simulation of boiling curve for contact angle = 10°. Vertical bars indicate the range of temporal fluctuations in heat flux.

For a contact angle = 10°, ΔT = 45K critical heat flux will result in Fig 6(a)-2. This is deduced based on a plot for heat flux (Fig 7). For wall temperatures around the critical heat flux condition, there are significant fluctuations in local surface heat flux due to bubble dynamics. The dry and wet regions change continuously. More vapor regions are formed during transient boiling (Fig 6(a)-3) with increasing wall superheat. In the case of contact angle = 10°, the vapor film is stable at the bottom boundary beyond ΔT = 60K. Thus, in this case, ΔT = 60K is the Leidenfrost point (LFP). The LFP is also the minimum heat flux point (Fig 7). The vapor film covers the entire bottom boundary leading to the film boiling regime (Fig 6(a)-4) at superheats beyond the LFP.

(ii) Hydrophobic surface:

For contact angles of 90 − 160°, it is observed that original vapor layer is still unstable at low superheats but it is increasingly stable for greater contact angles. As a result, the nucleate and transition boiling phases occur much earlier. For a contact angle of 160°, there is practically no transition boiling regime with a vapor film present in nearly all cases (Fig 6(d)-1 to 6(d)-4). The hydrophobic surface repels the liquid and stabilize the vapor film so that the small superheat causes Leidenfrost behavior. This is consistent with prior results [32].

#### 4.2.2 Boiling curve

**Fig 8:**
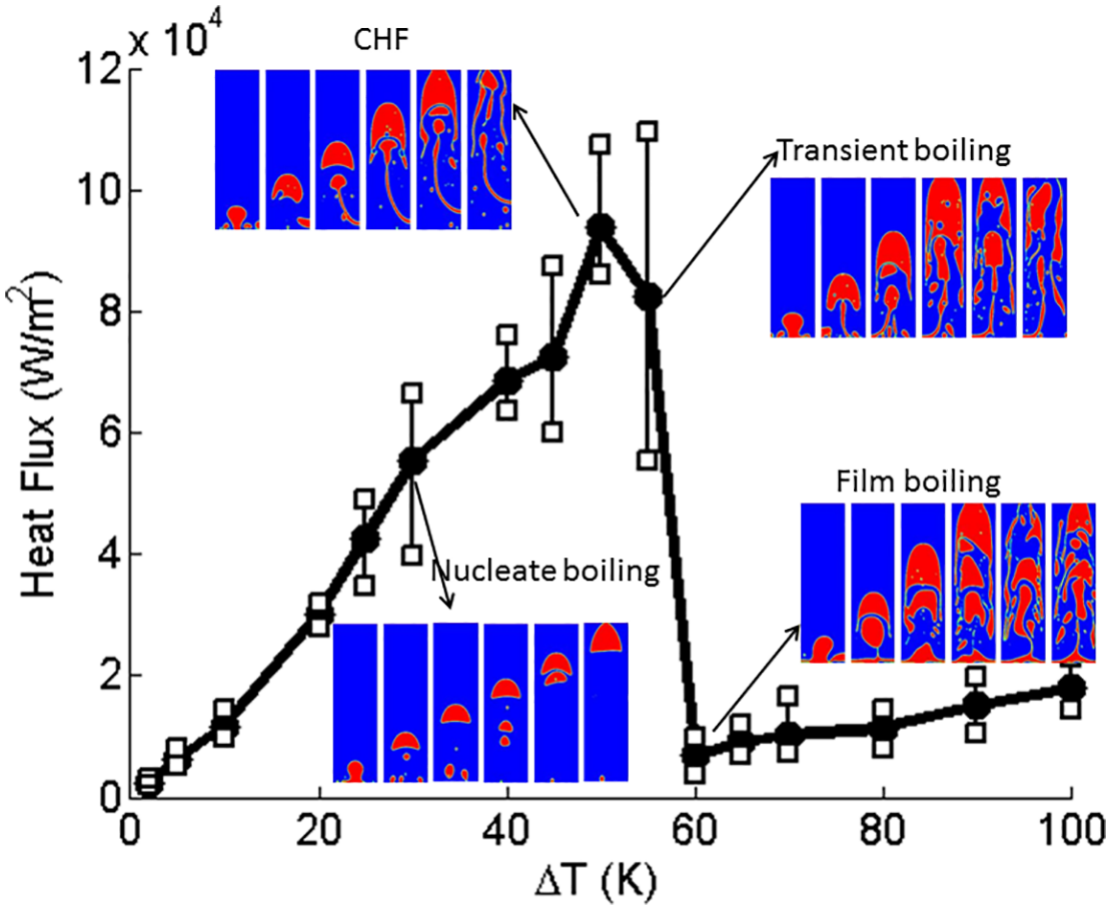
Two dimensional planar simulation of boiling curve for contact angle = 60°. Vertical bars indicate the range of temporal fluctuations in heat flux.

**Fig 9:**
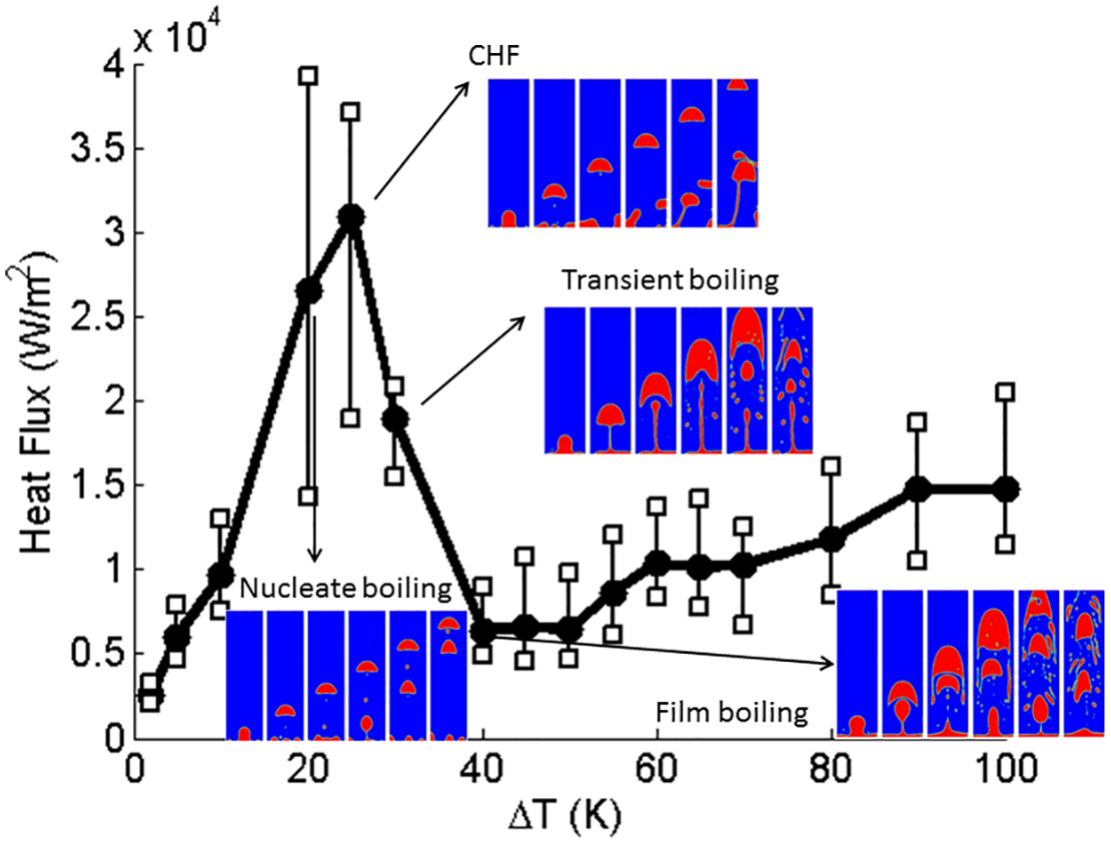
Two dimensional planar simulation of boiling curve for contact angle = 120°. Vertical bars indicate the range of temporal fluctuations in heat flux.

**Fig 10:**
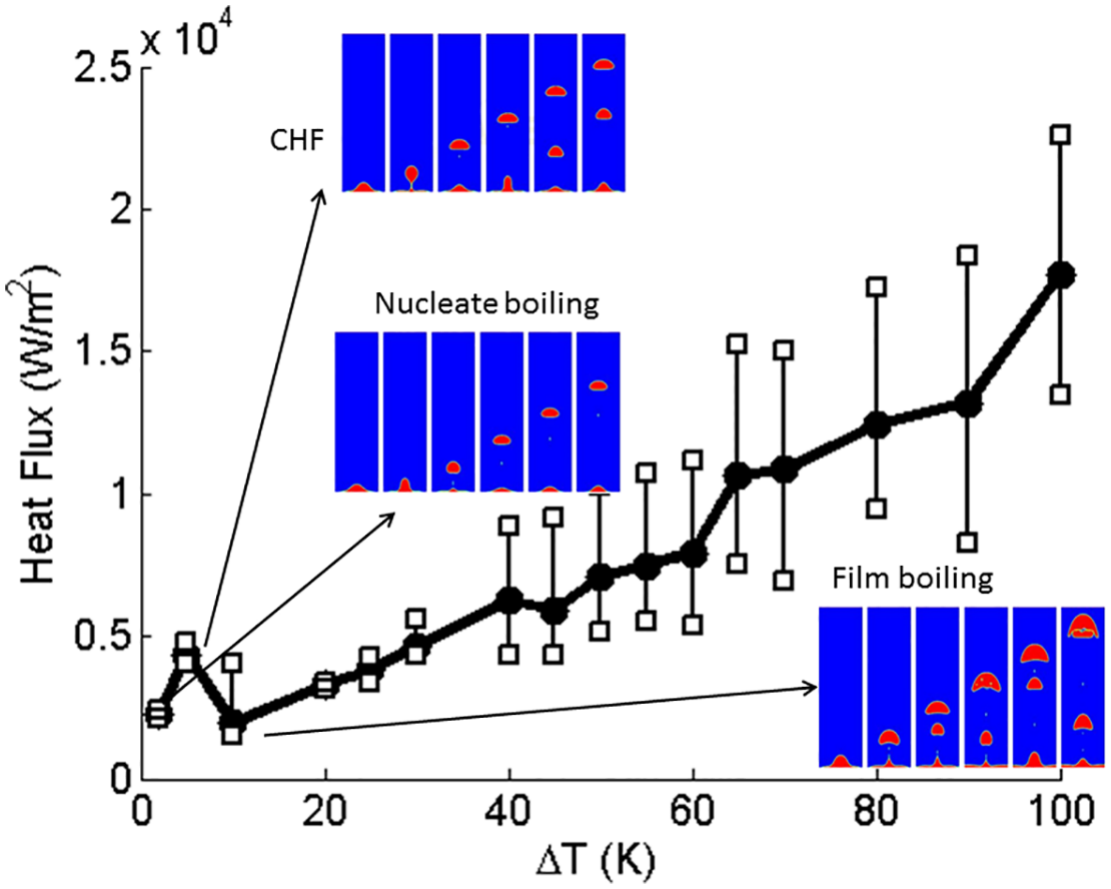
Two dimensional planar simulation of boiling curve for contact angle = 160°. Vertical bars indicate the range of temporal fluctuations in heat flux.

**Fig 11:**
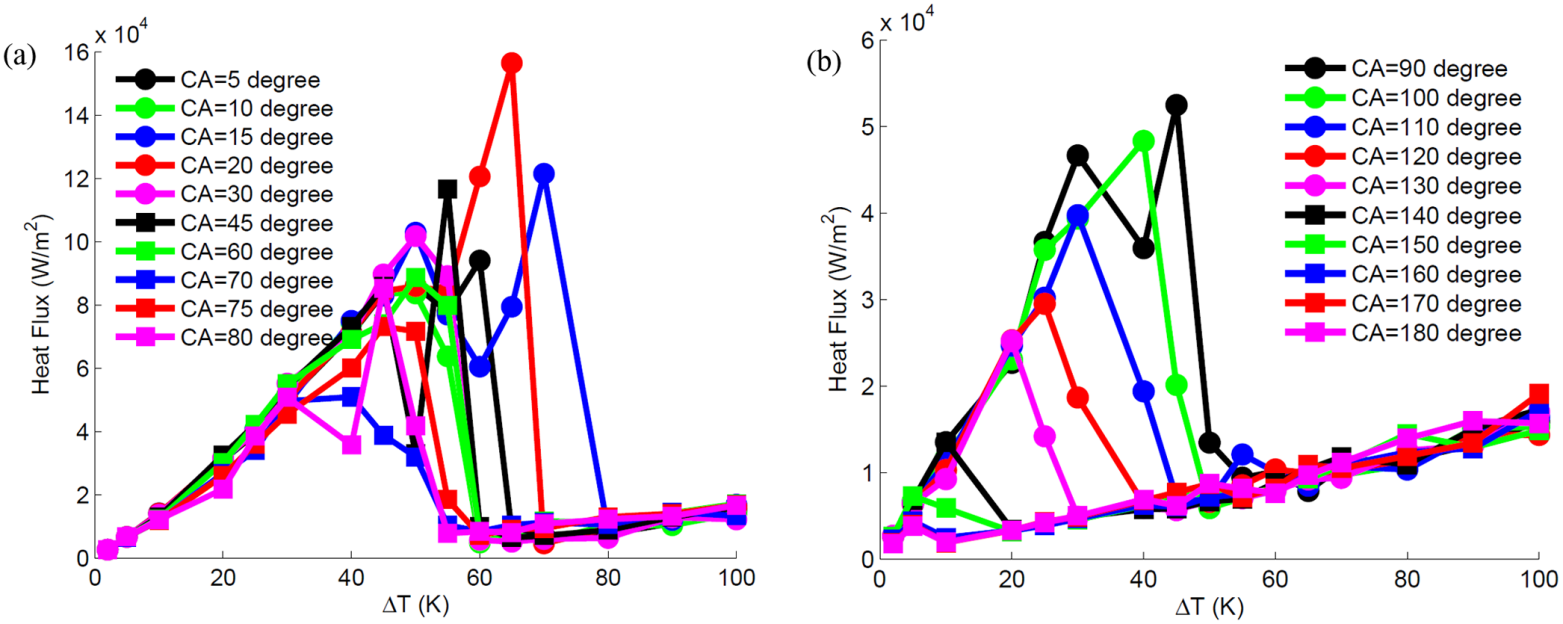
Boiling curves for (a) hydrophilic surfaces and (b) hydrophobic surfaces.

Boiling curves for the cases solved are shown in Figs 7–11. These figures show the effect of contact angle on the boiling curve. The heat flux is computed as

$$\begin{array}{c} q=k{\frac{dT}{dn}|}_{\text{near}\;\text{wall}} \end{array}$$ (16)

where *q* is the heat flux, *k* is the conductivity of the fluid, and *T* is the temperature. All key aspects of the boiling curve, namely nucleate boiling, critical heat flux condition, transition boiling, and film boiling are qualitatively captured in the calculated numerical results. Around the critical heat flux, the heat flux fluctuates significantly from instant to instant. Hence, the trends are more noisy. To emphasize this issue, the ranges of heat flux fluctuations are depicted in Figs 7–10 by error bars. Compared to the three dimensional simulation results, the heat flux of two dimensional is at the same order as the three dimensional heat flux. For contact angle = 60°, the CHF of boiling curve of the three dimensional case falls at ΔT = 65(K) and that of the two dimensional case falls at ΔT = 50(K). However, for contact angle = 120°, the boiling curve is almost identical.

#### 4.2.3 Critical heat flux

**Fig 12:**
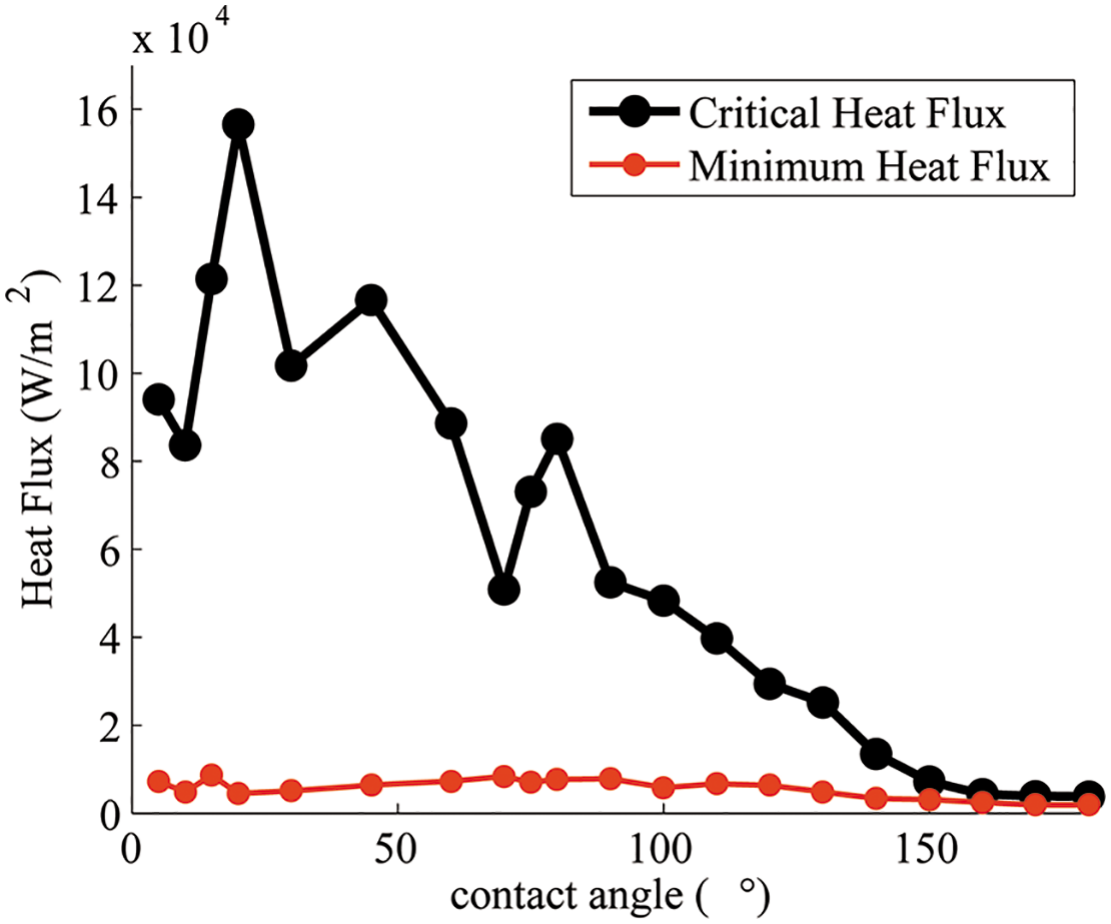
Maximum (critical) and minimum heat flux vs. contact angle.

**Fig 13:**
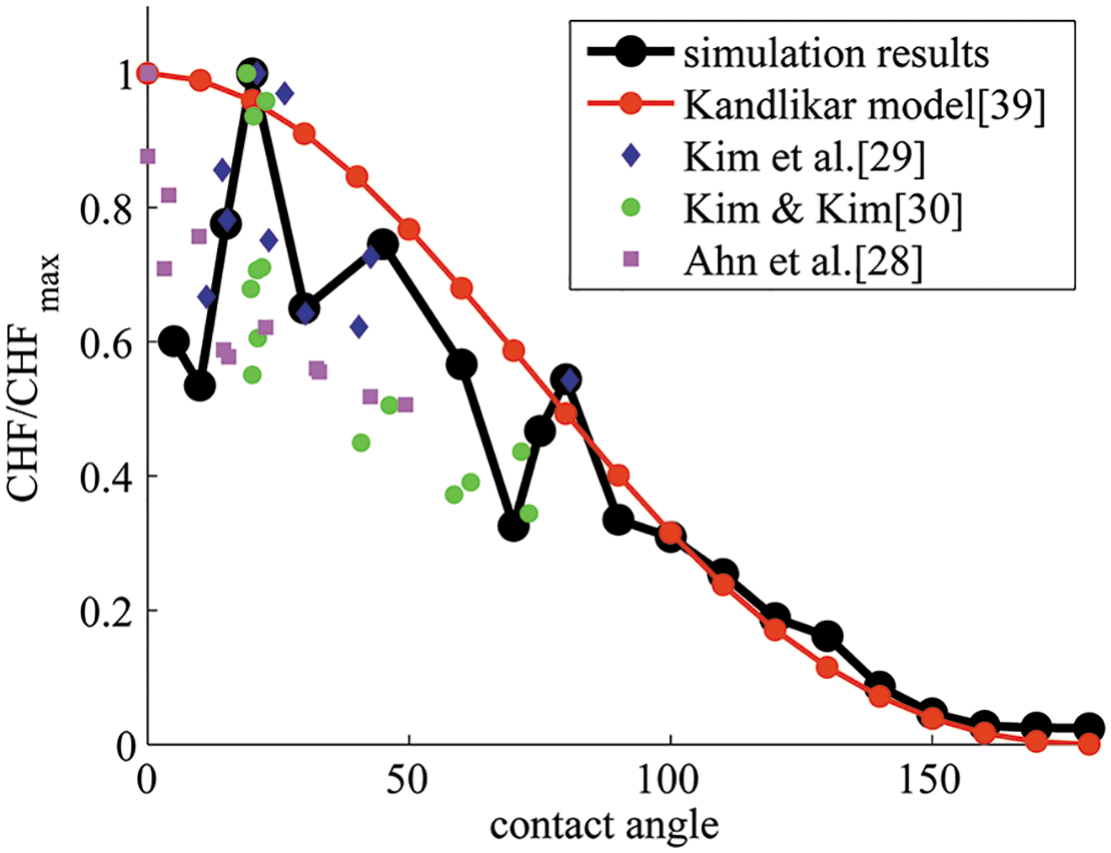
Critical heat flux normalized by the corresponding maximum value of CHF in simulation, model, and experimental data. Qualitative trends in CHF vs. contact angle in data from simulations, models, and experiments are compared.

Fig 12 shows the plot of critical heat flux at different contact angles. In order to enable comparison of the trend with models and experiments [33–35], normalized CHF data are plotted in Fig 13.

Many models of CHF have been developed [30, 36–41]. A commonly used model by Zuber [41] to calculate CHF is given by

$$\begin{array}{c} q_{max}^{\prime \prime }=0\text{.}131\rho_{v}h_{lv}{\left[\frac{\sigma (\rho_{l}-\rho_{v})g}{\rho_{v}^{2}}\right]}^{\frac{1}{4}}. \end{array}$$ (17)

Using properties of the working fluid in the simulations (*ρ*_*l*_ = 200 kg/m^3^, *ρ*_*v*_ = 5 kg/m^3^, *h*_*lv*_ = 1 × 10^4^ J/kg, and surface tension *σ* = 0.1 N/m), the critical heat flux is calculated to be 1.0891 × 10^5^ W/m^2^. This estimate is at the same order of magnitude as the maximum critical heat flux calculated in simulations. However, this correlation does not capture the effect of the contact angle on the CHF.

Kandlikar [30] proposed a model for CHF that accounts for the effect of surface wettability:

$$\begin{array}{c} q_{max}^{\prime \prime }=\kappa^{-\frac{1}{2}}\rho_{v}^{\frac{1}{2}}h_{lv}{\left[\sigma \left(\rho_{l}-\rho_{v}\right)g\right]}^{\frac{1}{4}} \end{array}$$ (18)

where *κ* is a surface-dependent parameter, which is large for a poor wetting surface but small for a strong wetting surface. It is given by:

$$\begin{array}{c} \kappa =\frac{{\left(1+cos\theta \right)}^{-2}}{16}{\left(\frac{2}{\pi }+\frac{\pi }{4}\left(1+cos\theta \right)cos\phi \right)}^{-1}, \end{array}$$ (19)

where *θ* is the contact angle and *ϕ* is the heater orientation angle relative to the horizontal. It has been found that our simulation results are quantitatively smaller than the values from the model proposed by Kandlikar [30], but qualitatively the trends are similar and comparable favorably for hydrophobic surfaces (Fig 13). For hydrophilic surfaces, the agreement between the model and numerical simulations is less favorable, the numerical data are noisy due to significant fluctuations in transient heat flux. Actually, similar non-monotonic data are obtained from experiments for hydrophilic surfaces as seen in Fig 13.

In comparison, greater fluctuation in CHF data is found to be related to liquid-vapor dynamics. CHF denotes the onset of transition boiling where there is greater tendency to form and break vapor film next to the wall. Since liquid prefers to remain in contact with a hydrophilic surface, there is greater tendency to destabilize the vapor film formation process. As a result, sometimes an asymmetric vapor film is formed (see Fig 6(a)-3 and 6(b)-3 and at other times, a symmetric vapor film (see Fig 6(a)-4 and 6(b)-4) is formed. The asymmetric vapor film case has a greater average heat transfer coefficient as compared to the symmetric case since a liquid of high conductivity is in contact with the wall over a greater surface area. These dynamics are dominant in the transition regime for hydrophilic walls until eventually the entire wall is covered with vapor.

Experimental data in the transition region of the boiling curve have been speculated to be “noisy” due to dynamic advancing and receding contact angles [9]. In this work we have static contact angles. From pooling boiling experiment [42], Ramanujapu and Dhir have shown that advancing and receding contact angles are within only ±5 deg of the static contact angle. For the first step of this study, the static contact angle model throughout the bubble evolution process is feasible. Yet, the fluctuation in the transition regime persists due to the presence of different unstable modes as discussed above. Some prior numerical results have shown that it can still captures key features of boiling without considering the pinning effect [43]. Thus, at the first step in this work, the pinning effect is not included in the simulations while being able to capture the primary mechanism.

#### 4.2.4 Leidenfrost point

The Leidenfrost point characterizes the onset of stable film boiling and minimum heat flux. Fig 12 shows the plot of minimum heat flux at different contact angles. Berenson’s minimum heat flux model is given by [44]:

$$\begin{array}{c} q_{min}^{\prime \prime }=0\text{.}09\rho_{v}h_{lv}{\left[\frac{g\sigma (\rho_{l}-\rho_{v})}{{\left(\rho_{v}+\rho_{l}\right)}^{2}}\right]}^{\frac{1}{4}}. \end{array}$$ (20)

From the fluid properties used in simulations, minimum heat flux is found to be 1.168 × 10^3^ W/m^2^, which is close to our simulation values.

**Fig 14:**
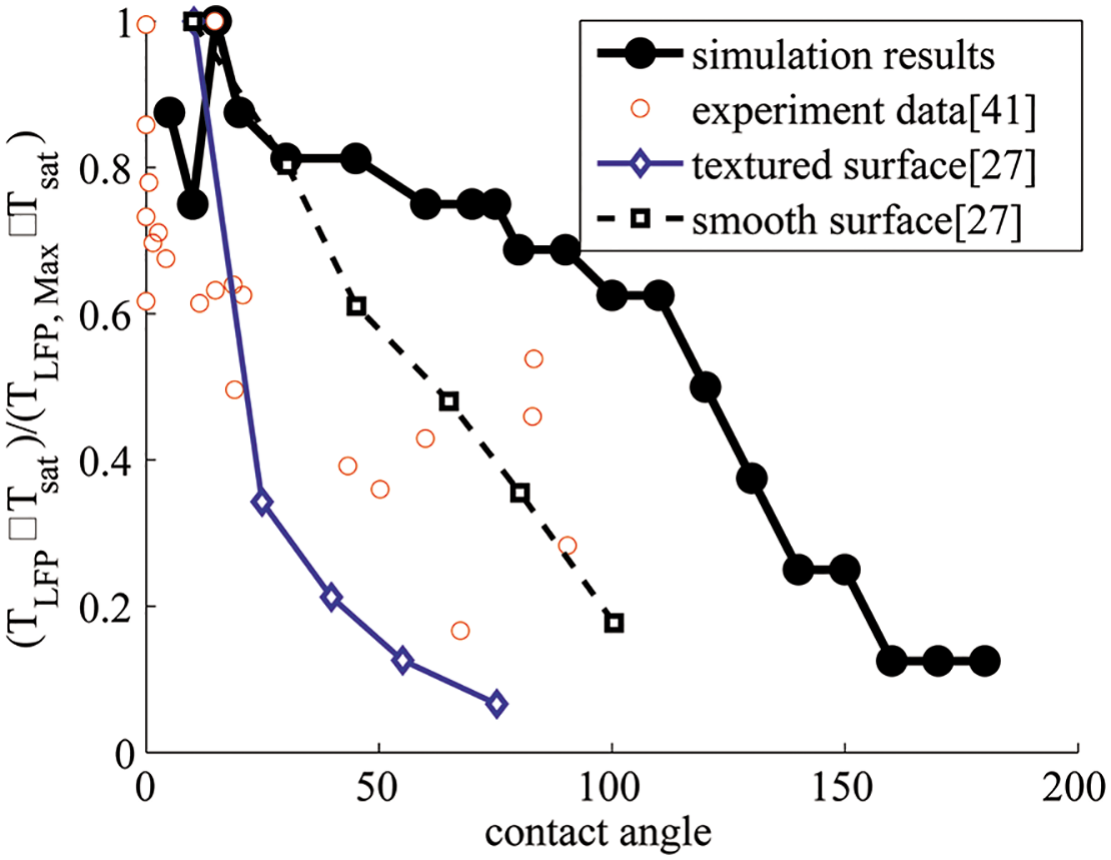
Comparison of the trends of Leidenfrost point vs. contact angle calculated from simulations and experimental data. The Leidenfrost temperature is normalized by the corresponding maximum value of Leidenfrost temperature in simulation and experimental data.

Fig 14 shows the plot of the Leidenfrost point (LFP) at different contact angles determined from our simulation results. The trend from simulations is compared to experimental data by plotting normalized values of the Leidenfrost temperature [32, 45]. In both cases, the LFP decreases with increasing contact angle; however, experimental data show a sharper decrease of LFP with contact angles in the hydrophilic regime when compared to simulations. This may be due to the effect of surface roughness. Consider two experimental cases—“textured surface” and “smooth surface” from a prior work [32], as shown in Fig 14. Smoother experimental surfaces (experimental surfaces are not perfectly smooth) are found to show a shallower plot for Leidenfrost temperature due to fewer nucleation sites. Simulation data in this work are for smooth surfaces without any nucleation models. In this case, it is expected that the vapor film will be less stable for hydrophilic walls as indicated by a shallower plot for Leidenfrost temperature in the hydrophilic regime. This suggests that smooth wall simulations may provide a baseline limiting case for the development of suitable models for LFP.

#### 4.2.5 Temperature field

**Fig 15:**
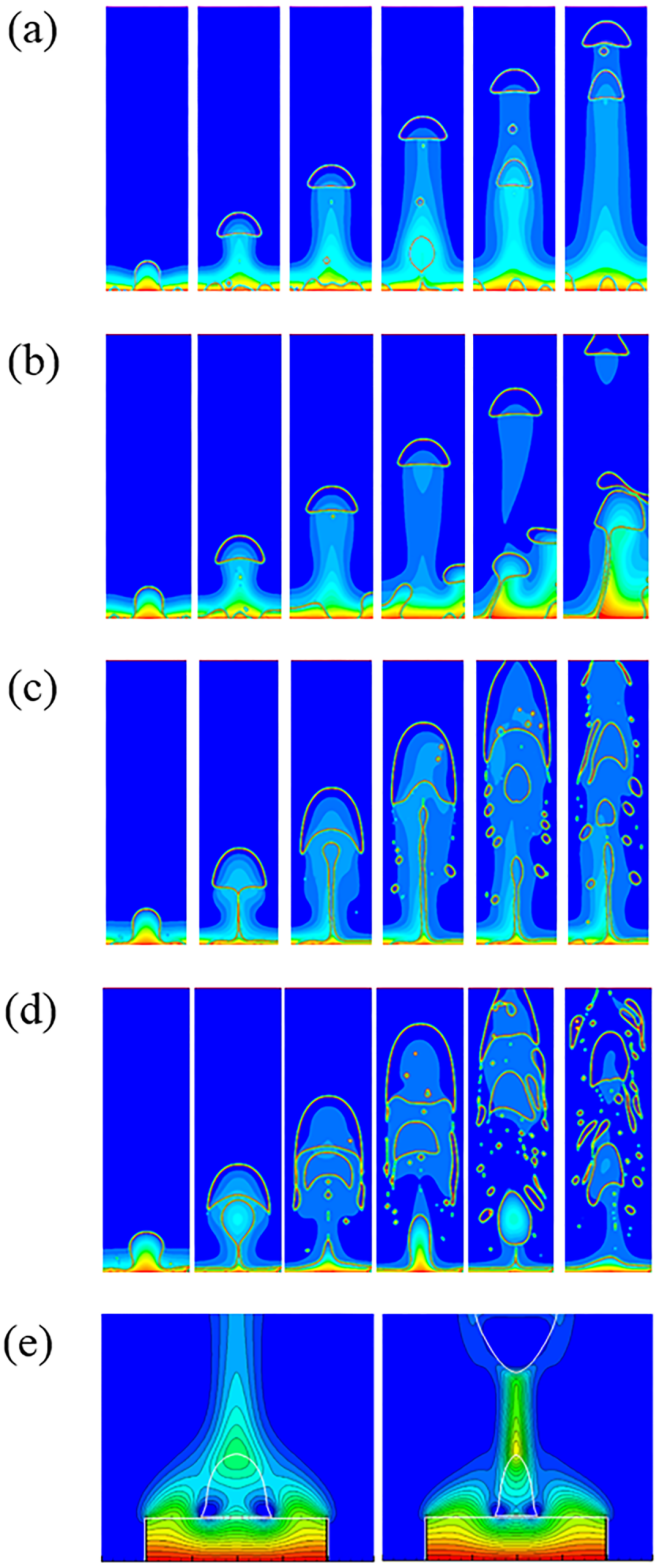
Temperature fields at contact angle = 120° for (a)nucleate boiling, (b)CHF, (c)transient boiling, (d)LFP, and (e)LBM results of pool boiling process on a hydrophobic surface [16].

Following experimental results and Boltzmann simulation work [11, 12, 15, 16, 22], in this research the liquid-vapor interface is not constrained to be at the saturation temperature. Instead, an interfacial heat flux exchange model is adapted. Fig 15a–15d show temperature profiles with a gradient of temperatures across the liquid-vapor interface which is similar to prior LBM work [15, 16]. Fig 15e is the temperature contour result of vapor bubble within the Lattice Boltzmann method which alo shows a higher temperature inside the bubble and gradient interface temperature. From experiment and other related research [11, 12, 22], the temperature at the interface as well as inside the bubble is higher than *T*_*sat*_ for growing bubble on the heating surface which is in agreement with our results. The corresponding boiling curves are found to have similar trends as expected. It was found that the imposition of saturation temperatures on the liquid-vapor interface destabilized the formation of the vapor film. Consequently, the transition to film boiling in the boiling curve was significantly delayed or not observed for the parameters we tested. A detailed investigation is not within the scope of this work but is warranted in future.

## 5 Conclusion

In this work, numerical simulations of evolving liquid-vapor interfaces during pool boiling on a horizontal smooth surface have been performed to study the surface wettability effect and related dynamics. Instead of a saturated temperature constrained interface, an interfacial heat flux exchange model at the interface is adapted. The simulation results based on the volume-of-fluid method and static contact angle model have been carried out and compared with some prior theoretical and experimental predictions, demonstrating good agreement. The effect of surface contact angle and superheat on the complete boiling heat transfer curve is obtained for the first time and the corresponding dynamics has been qualitatively captured. It is verified this approach can be used for investigating boiling phenomena and providing more physical insights into the corresponding dynamics. In addition, it can provide some guidance for more time consuming three dimensional cylindrical numerical simulations. In a near future, specific boiling regime will be focused and more physics behind the dynamics can be assured.

## Supporting information

S1 Data Set Data set for boiling curve at different contact angle and superheats. (XLSX)
